# Gap balancing versus measured resection in primary total knee arthroplasty: A retrospective cohort study protocol

**DOI:** 10.1097/MD.0000000000020017

**Published:** 2020-05-15

**Authors:** Guo-rong She, Zhen-gang Zha

**Affiliations:** Department of Bone and Joint Surgery, The First Affiliated Hospital of Jinan University, Guangzhou 510630, Guangdong Province, China.

**Keywords:** gap balancing, measured resection, protocol, total knee arthroplasty

## Abstract

**Background:**

Recently, controversy still exists regarding the clinical effects of measured resection or gap-balancing technique in total knee arthroplasty (TKA). The objective of this retrospective study was to compare the clinical outcomes of conventional measured resection technique and computer-assisted gap-balancing technique in TKA.

**Methods:**

Strengthening the Reporting of Observational studies in Epidemiology checklist. Patients underwent primary TKA by a single surgeon between 2014 and 2016 were reviewed. This study was approved by the institutional review board in our hospital and was registered in the Research Registry. Outcome measures included surgical time, intraoperative complications, patient satisfaction, Oxford Knee Score, range of motion, postoperative complications, and revision.

**Results:**

This study had limited inclusion and exclusion criteria and a well-controlled intervention.

**Conclusion:**

We were able to directly compare the outcomes of measured resection versus gap-balancing techniques and might reveal a better technique in TKA.

**Trial registration:**

This study protocol was registered in Research Registry (researchregistry5441).

## 1 Introduction

Total knee arthroplasty (TKA) may be the best treatment for the elderly patients with end-stage osteoarthritis.^[1,2]^ Given the aging population, combined with the exponential increase in obesity, the incidence of osteoarthritis and subsequent demand for TKA is increasing.^[3]^ The success of TKA depends on restoration of limb alignment, accurate implant position, and optimal gap balancing. Malpositioning of the femoral or tibial component can lead to early loosening, increased polyethylene wear, and poor patellar tracking.^[4]^

To achieve a balanced knee, 2 distinct methods of knee prosthesis implantation have been described: measured resection and gap balancing, or balanced resection. The first technique was performed in conventional total knee arthroplasty, where femoral and tibial cuts were made first. These resections were based on bony anatomical landmarks and were followed by soft-tissue balancing to achieve rectangular flexion and extension gaps. This technique was noted to cause less reduction in the postoperative joint line.^[5]^ However, the distorted landmarks in a deformed arthritic knee and the fact that soft-tissue releases often affect flexion and extension gaps asymmetrically, contribute to inaccuracies in restoring normal balance.^[4,6]^ The gap equalization technique in computer-aided surgery precluded the use of bony landmarks and instead relied on optimal soft-tissue tensioning before the final femoral resection. The use of computer navigation increased the accuracy of the crucial proximal tibial cut and quantified soft-tissue values in the gap-balancing technique.^[7]^ In addition, computer-aided navigation offered the capability to track independent lengthening and shortening of the collateral ligaments, which are the major stabilizers in 30° to 60° of flexion.^[8]^ As the gap-balancing technique is dependent on integrity of the collateral ligaments, this capability of computer-aided surgery facilitates the selection of a femoral component that properly tenses these ligaments through a full range of motion.^[7]^

Recently, controversy still exists regarding the clinical effects of measured resection or gap-balancing technique in TKA. Due to a lack of direct comparison between the clinical outcomes of these 2 techniques in current literature, uncertainty remains regarding the superiority of either method. The purpose of this study was to evaluate:

(1) implant survivorship,
(2) patient outcomes, and
(3) complications in patients who underwent TKA with either measured resection or gap-balancing technique by the same experienced surgeon.

The hypothesis was that the computer-assisted gap-balancing technique would achieve better functional scores and fewer complications as compared to the measured resection technique in conventional TKA.

## 2 Materials and methods

This study was performed and reported in accordance with the Strengthening the Reporting of Observational studies in Epidemiology checklist.

### 2.1 Patients

We reviewed patients who underwent primary TKA at our academic center from 2014 to 2016. This retrospective cohort study was approved by the institutional review board in our hospital (PRS2020093) and was registered in the Research Registry (researchregistry5441). The inclusion criterion were set as follows:

(1) osteoarthritis of the knee requiring primary TKA during hospitalization;
(2) patients that were over 18 years old and could cooperate with us for treatment and postoperative observation;
(3) full demographic and follow-up data.

Exclusion criteria included prior knee surgery, if the patient was pregnant or trying to become pregnant, cognitive impairment, a history of alcoholism, or if the patient was to undergo bilateral, simultaneous TKA.

### 2.2 Techniques

All TKAs in this study were carried out by a single senior surgeon. A tourniquet was used in all cases, general anesthesia was administered to each patient before incision, and the operative knee was prepared and draped in a conventional sterile fashion. The implants utilized were beaded periapatite-coated femoral, tibial, and patellar components (Triathlon Total Knee System; Stryker Orthopaedics, Mahwah, NJ). The specific technical details of the 2 groups were as follows:

#### 2.2.1 Measured resection

After adequate exposure of the knee, femur is drilled and internal femoral alignment rod is introduced into the intramedullary canal, followed by distal cutting block with preset parameters. Distal femoral cut is performed. Posterior referencing cutting block is utilized to identify the potential component size. Appropriate 4-in-1 resection block is utilized to perform anterior, posterior, and chamfer bone cuts. Extra-medullary guide is used to resect a predetermined thickness of proximal tibia. Previously decided trial sizes and polyethylene are introduced and the knee is evaluated for tracking stability in the anterior–posterior and varus and valgus planes for balance.

#### 2.2.2 Gap nalancing

Distal femoral and proximal tibial cuts are performed so that the cut surfaces are parallel. Subsequently, the knee is brought to extension and the appropriate sized spacer block is introduced. Medial and lateral balance is evaluated, appropriate soft-tissue releases are made, and extension tension is recorded. After that, the knee is brought into flexion, sizer balancer is introduced and tensed to the same tension as the noted extension tension, medial and lateral balance is evaluated, and appropriate soft-tissue releases are made if necessary. The remaining femoral cuts are subsequently made.

### 2.3 Postoperative care

Postoperative drainage lasted 1 to 2 days until flow volume was less than 30 ml. All patients received the same standardized postoperative multimodal pain protocol, with 4 doses of 1 g of acetaminophen, 2 doses of celecoxib 200 mg, and morphine (first 48 h) or tramadol (after 48 h) for pain exacerbations. All patients underwent the same postoperative rehabilitation program, with partial weight bearing with the use of crutches for the first postoperative day and active range of movement exercises.

### 2.4 Outcome evaluation

The patient demographics, American Society of Anesthesiologists Score grade, and body mass index were recorded retrospectively from their electronic patient notes. Outcome measures included surgical time, intraoperative complications, patient satisfaction, Oxford Knee Score (OKS), range of motion, postoperative complications, and revision. Surgical time and intraoperative complications were obtained from our hospital database, as well as electronic and paper records. The OKS, range of motion, postoperative complications, and revision were obtained both before and after surgery at a minimum of 3 years postoperatively. Subjects were asked to complete an OKS questionnaire and rate their satisfaction with their TKA. The OKS consists of 12 questions assessed on a Likert scale with values from 0 to 4, a summative score is then calculated where 48 is the best possible score (least symptomatic) and 0 is the worst possible score (most symptomatic). Patient satisfaction was assessed by asking the question “How satisfied are you with your operated knee?” 1 year after surgery. The response was recorded using a 5-point Likert scale: very satisfied, satisfied, neutral, unsatisfied, and very unsatisfied. Patients who recorded very satisfied or satisfied were classified as satisfied. For patients who were not seen recently, the scores were obtained via telephone. Postoperative complications and revision procedures were documented during routine collection of follow-up data. All data were independently verified by a detailed review of hospital operative reports, anesthesia records, and clinical records. Data were abstracted by 1 of 2 research personnel blinded to patient group and study aim.

### 2.5 Statistical analysis

Statistical analysis was performed using Statistical Package for Social Sciences version 20.0 (IBM Corporation, Armonk, NY). Parametric and non-parametric tests were used as appropriate to assess continuous variables for significant differences between groups. A Student's *t* test was used to compare linear variables between groups. Dichotomous variables were assessed using a Chi-square test. Multivariate linear and regression analyses were used to identify independent predictors of outcome (postoperative OKS). A *P*-value of <.05 was defined as statistical significance. A post-hoc power calculation was performed for the OKS: with 44 patients in the gap-balancing group and 69 in the measured resection group and a defined minimal clinically important difference of 5 points with a standard deviation of 9 and an alpha 0.05 achieved a power of 0.81.

## 3 Result

The results will be shown in Tables 1 and 2.

**Table 1:**
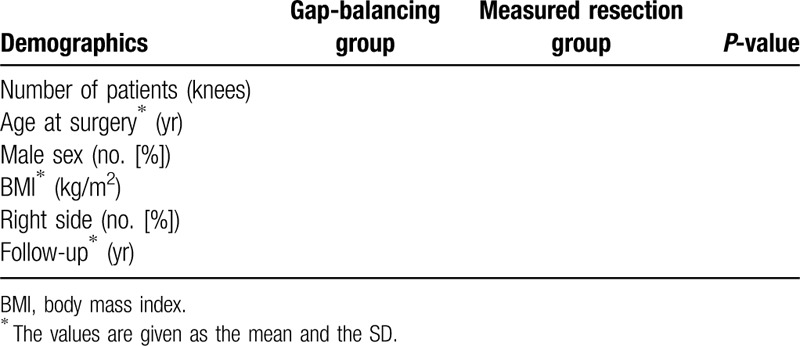
Patient baseline demographics.

**Table 2:**
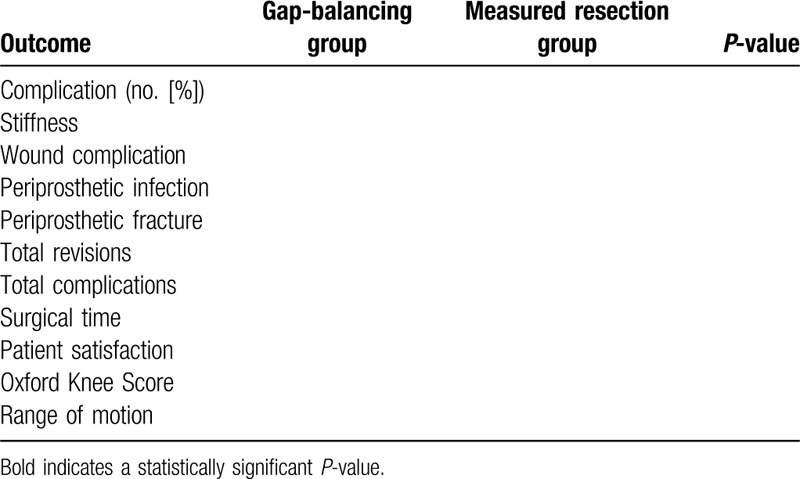
The outcomes in the 2 groups.

## 4 Discussion

The primary goal of TKA is restoration of mechanical axis and soft-tissue balance. Improper restoration may lead to poor functional outcome and premature prosthesis loosening and subsequent failure. Computer navigation enables precise femoral and tibial cuts and controlled soft-tissue release. Mechanical alignment and soft-tissue balance is closely interlinked and can be corrected by soft-tissue releases and by altering the proximal tibial and distal femoral cuts. The 2 common techniques used are measured resection and gap-balancing techniques.^[9,10,11,12,13]^

Some surgeons favour gap-balancing technique, as it provides more consistent soft-tissue tension in TKA. The measured resection technique can be inaccurate lead to improper alignment of femoral component, because of wide variation in the anatomy of bony landmarks (epicondyles, the anteroposterior line, and posterior condyles) when making the femoral cuts.^[14,15,16,17,18,19]^ Others consider measured resection as an outdated technique in knee arthroplasty.^[12]^ Nonetheless, a combination of these bony landmarks helps to minimize the error of component malposition owing to anatomic variation.

Limitations of this study included single surgeon practice, single implant manufacturer, and single implant model utilized, lack of patient randomization, and no advanced imaging (computed tomography scan) for accurate preoperative and postoperative measurements. In addition, the limitations of our study also included those inherent in any retrospective cohort study, including the possibility of selection or observational bias. This study also did not address long-term follow-up (10 years) as our study relied on electronic medical records kept since 2014. The authors recognized that longer-term follow-up was critical in determining the differences of gap balancing and measured resection technique in TKA. Despite the above-mentioned limitations of this study, we were able to directly compare the outcomes of measured resection versus gap-balancing techniques and might reveal a better technique in TKA.

## Author contributions

Guo-rong She planned the study design and wrote the study protocol. Guo-rong She and Zhen-gang Zha reviewed the study protocol. Guo-rong She and Zhen-gang Zha will recruit participants and collect data. Guo-rong She wrote the manuscript. All of the authors have read, commented on, and contributed to the submitted manuscript.
